# *YTHDF1* rs6090311 A>G polymorphism reduces Hepatoblastoma risk: Evidence from a seven-center case-control study

**DOI:** 10.7150/jca.46120

**Published:** 2020-06-28

**Authors:** Zhendong Luo, Guoyuan Li, Mi Wang, Jinhong Zhu, Zhonghua Yang, Yong Li, Jiao Zhang, Yijuan Xin, Suhong Li, Li Li, Zhenjian Zhuo, Jing He

**Affiliations:** 1 Department of Pediatric Surgery, Guangzhou Institute of Pediatrics, Guangdong Provincial Key Laboratory of Research in Structural Birth Defect Disease, Guangzhou Women and Children's Medical Center, Guangzhou Medical University, Guangzhou 510623, Guangdong, China.; 2 Department of Clinical Laboratory, Biobank, Harbin Medical University Cancer Hospital, Harbin 150040, Heilongjiang, China.; 3 Department of Pediatric Surgery, Shengjing Hospital of China Medical University, Shenyang 110004, Liaoning, China.; 4 Department of Pediatric Surgery, Hunan Children's Hospital, Changsha 410004, Hunan, China.; 5 Department of Pediatric Surgery, the First Affiliated Hospital of Zhengzhou University, Zhengzhou 450052, Henan, China.; 6 Clinical Laboratory Medicine Center of PLA, Xijing Hospital, Air Force Medical University, Xi'an 710032, Shaanxi, China.; 7 Department of Pathology, Children Hospital and Women Health Center of Shanxi, Taiyuan 030013, Shannxi, China.; 8 Kunming Key Laboratory of Children Infection and Immunity, Yunnan Key Laboratory of Children's Major Disease Research, Yunnan Institute of Pediatrics Research, Yunnan Medical Center for Pediatric Diseases, Kunming Children's Hospital, Kunming 650228, Yunnan, China.

**Keywords:** hepatoblastoma, risk, *YTHDF1*, polymorphism, case-control study

## Abstract

Various factors modulate the risk of hepatoblastoma. In this study, we aimed to investigate whether single nucleotide polymorphisms (SNPs) in the *YTHDF1* gene could predispose to hepatoblastoma. We used TaqMan assay to genotype two *YTHDF1* SNPs (rs6011668 C>T and rs6090311 A>G) in a Chinese population composed of 313 subjects with hepatoblastoma and 1446 controls from seven hospitals. We then evaluated the associations of these two SNPs with hepatoblastoma risk using unconditional logistic regression. We found that rs6090311 G allele exhibited a significant association with decreased hepatoblastoma risk [AG vs. AA: adjusted odds ratio (OR)=0.75; 95% confidence interval (CI)=0.58-0.98, *P*=0.033; AG/GG vs. AA: adjusted OR=0.76, 95% CI=0.59-0.97, *P*=0.029]. Furthermore, the combined analysis of protective genotypes revealed that subjects carrying two protective genotypes were less likely to have hepatoblastoma than those with 0-1 protective genotypes (adjusted OR=0.75, 95% CI=0.59-0.96, *P*=0.022). Subjects ≥17 months of age had decreased hepatoblastoma risk, in case that they carried rs6090311 AG/GG (adjusted OR=0.63, 95% CI=0.44-0.91, *P*=0.012), or two protective genotypes (adjusted OR=0.63, 95% CI=0.44-0.91, *P*=0.012). False-positive report probability analysis validated the reliability of the significant results. Preliminary functional annotations revealed that rs6090311 G was correlated with decreased expression of its surrounding genes in the expression quantitative trait locus (eQTL) analysis. In conclusion, our results indicate that the rs6090311 A>G in the *YTHDF1* gene is related to decreased hepatoblastoma risk.

## Introduction

Hepatoblastoma is the most prevalent liver tumor in childhood, accounting for up to 80% of all pediatric liver tumor cases 1, 2. The incidence of hepatoblastoma is low, with about 1.5 cases per million annually 3. The occurrence rate of hepatoblastoma is about 1.1 cases in a million children in China 4. The five-year survival rate for hepatoblastoma is about 80% 5. However, the survival rate may sharply decrease to 30-40% in the high-risk hepatoblastoma 6-8.

Hepatoblastoma mainly derives from abnormal differentiation of hepatocyte precursors of an epithelial lineage 9. Fibrosing diseases, hepatitis B virus, or cirrhosis are tightly associated with hepatocellular carcinoma, but not hepatoblastoma 10-12. Alternatively, it has been well documented that hepatoblastoma is associated with prematurity, low birth weight, trisomy 18, familial adenomatous polyposis, and Beckwith-Wiedemann syndrome 13-16. Moreover, parental tobacco use, loss of heterozygosity (LOH) at 11p15, and mutations in genes (such as* FGFR3*,* APC*, and *β-catenin*) have also been observed to significantly associate with hepatoblastoma risk in some studies, despite conflicting reports 17-22. Advances in the etiology of hepatoblastoma have been far lagged behind, when compared to those more common pediatric cancers. Only a few case-control studies have investigated hepatoblastoma susceptibility genetic variants to date. Besides, the sample size of these studies was relatively small, with less than 200 cases 23, 24. Previously, we conducted three extensive epidemiology investigations to identify the genetic variants for hepatoblastoma 25-27.

N6-methyladenosine (m^6^A) modification is the most recurrent internal modification for mRNAs/ncRNAs 28, 29. m^6^A modification is dynamically deposited, removed, and recognized by m^6^A methyltransferases (“writers”), demethylases (“erasers”), and m^6^A-specific binding proteins (“readers”), respectively 30. m^6^A “readers” include the YTH-family proteins YTHDF1-3, YTHDC1-2, and insulin-like growth factor 2 mRNA-binding proteins IGF2BP1-3. YTHDF1 interacts with translation initiation factors eIF3 and eIF4A3 to facilitate the translation process of m^6^A-modified mRNAs in the cytosol 31.

Multiple evidence has suggested the implicated of m^6^A regulators in the carcinogenesis, including METTL3, METTL14, ALKBH5, FTO, and YTHDF2 32-40. However, the significance of YTHDF1 in cancer is largely unknown. Given the rarity of hepatoblastoma, most of the existing epidemiology studies suffer from small sample size. With this in mind, we performed a relatively large case-control study to identify more hepatoblastoma susceptibility genetic variants in the *YTHDF1* gene.

## Materials and Methods

### Sample selection

The characteristics information of subjects were listed in **Table S1**. Overall, 313 patients with histologically confirmed hepatoblastoma were recruited from seven hospitals located in seven cities of China (Guangzhou, Xi'an, Zhengzhou, Changsha, Kunming, Shenyang, Taiyuan). Meanwhile, we recruited 1446 geographically matched control participants without a history of cancer or other diseases 41. Blood samples were obtained from all participants, along with informed consents. The study was approved by the institutional review board of all the participating hospitals.

### Polymorphism selection and genotyping

Through extensively mining the dbSNP and SNPinfo databases, we identified two potentially functional polymorphisms (rs6011668 C>T and rs6090311 A>G) in the *YTHDF1* gene. To be specific, the following criteria were adopted to choose potentially functional polymorphisms: SNPs located in the 5' flanking region, 5' untranslated region, 3' untranslated region, and exon of *YTHDF1* gene. Both the two selected SNPs (rs6011668 C>T and rs6090311 A>G) are located in the transcription factor binding sites (TFBS). There is no significant linkage disequilibrium (LD) (R^2^<0.8) among these two selected SNPs of *YTHDF1* gene (R^2^=0.094 between rs6011668 C>T and rs6090311 A>G) (**Figure S1**). Genomic DNA was prepared from peripheral blood, as previously described 42. SNP genotyping was implemented using the TaqMan assay according to the manufacturer's instructions (Applied Biosystems) 43. Internal negative controls (water) were used to ensure genotyping accuracy. About 10% of all samples were randomly selected to be genotyped again. The concordant rate of these duplicate samples was 100%.

### Statistical analysis

The χ^2^ test and the student's *t*-test was used to assess the differences in the distribution of gender and age, respectively, between cases and controls. Each SNP was tested in controls to ensure the fitting with Hardy-Weinberg equilibrium (HWE) using a χ^2^ test. Multiple logistic regression analysis was performed to evaluate an association between SNPs and hepatoblastoma risk. The odds ratios (ORs), 95% confidence intervals (CIs), and *P* values were calculated. Data were further stratified by age, gender, and clinical stages. False-positive report probability (FPRP) analysis was conducted to assess noteworthy associations by the means as described elsewhere 44, 45. Expression quantitative trait loci (eQTL) analysis using GTEx portal web site (http://www.gtexportal.org/home/) was conducted to determine the correlation between the SNPs and expression levels of nearby genes 46. The level of significance was set at 0.05. All statistics were performed using SAS 9.1 (SAS Institute, Cary, NC).

## Results

### Association between *YTHDF1* polymorphisms and hepatoblastoma susceptibility

We successfully genotyped the two polymorphisms in 313 cases and 1444 controls. The genotype frequencies of the two SNPs are listed in **Table 1**. Both of the two SNPs were in agreement with the HWE in controls (*P*_HWE_=0.882 for rs6011668 C>T; *P*_HWE_=0.240 for rs6090311 A>G). No significant association was found between the rs6011668 C>T polymorphism and hepatoblastoma susceptibility. We found that the rs6090311 G allele was significantly associated with protection against hepatoblastoma (AG vs. AA: adjusted OR=0.75, 95% CI=0.58-0.98, *P*=0.033; AG/GG vs. AA: adjusted OR=0.76, 95% CI=0.59-0.97, *P*=0.029). We next sought to determine whether the combination of rs6011668 C>T and rs6090311 A>G have a more powerful effect on hepatoblastoma risk than either one alone. The rs6011668 CC/CT and rs6090311 AG/GG were treated as protective genotypes. Subjects carrying two protective genotypes showed significantly decreased hepatoblastoma risk when compared with those with 0-1 protective genotypes (adjusted OR=0.75, 95% CI=0.59-0.96, *P*=0.022).

### Stratification analysis

To assess whether these variants influence susceptibility in specific subgroups, we performed a stratification analysis regarding age, gender, and clinical stages (**Table 2**). When compared to the AA genotype, the protective effect of rs6090311 AG/GG genotypes was more evident among children ≥17 months of age (adjusted OR=0.63, 95% CI=0.44-0.91, *P*=0.012). Besides, the combined analysis also demonstrated that the carriers with two protective genotypes had a significantly decreased hepatoblastoma risk among children aged 17 months and older (adjusted OR=0.63, 95% CI=0.44-0.91, *P*=0.012).

### False-positive report probability (FPRP) analysis

FPRP analysis was carried out to interrogate our significant findings (**Table 3**). The threshold for FPRP was preset as 0.2. At the prior probability level of 0.1, findings for 2 vs. 0-1 protective genotypes remained noteworthy.

### Expression quantitative trait loci (eQTL) analyses

**Figure 1:**
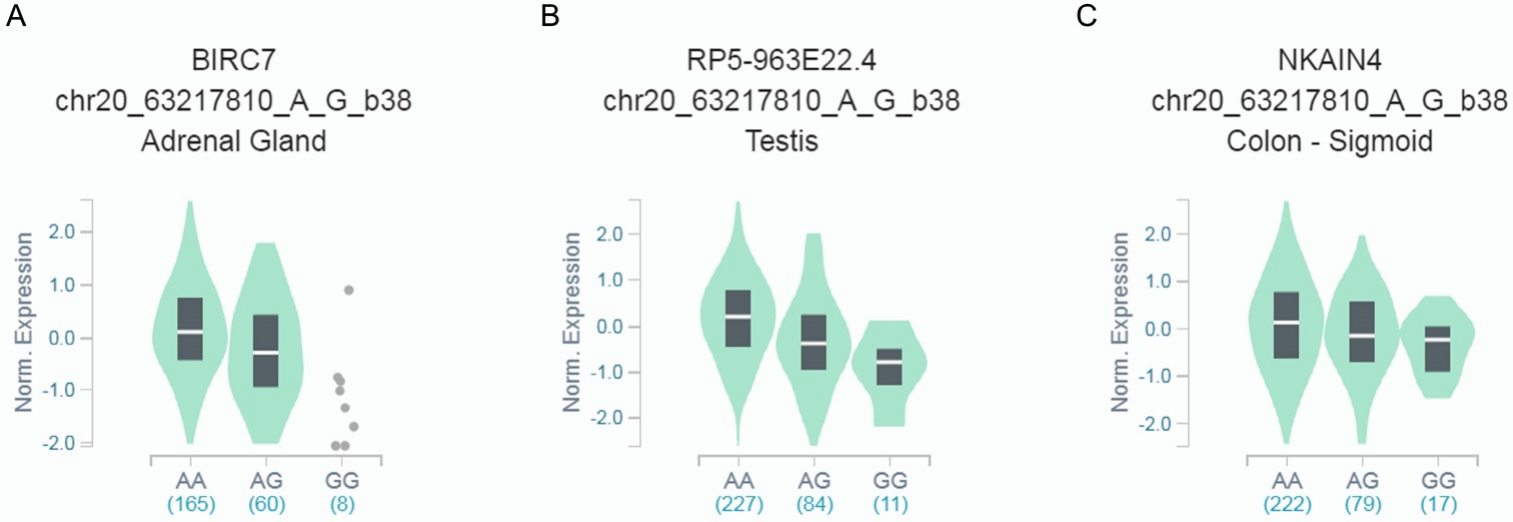
**Functional implication of rs6090311 polymorphism based on the public database GTEx Portal**. (A) The genotype of rs6090311 and expression of *BIRC7* gene in adrenal gland; (B) The genotype of rs6090311 and expression of *RP5-963E22.4* gene in testis; (C) The genotype of rs6090311 and expression of *NKAIN4* gene in colon-sigmoid.

We further explored whether rs6090311 A>G could influence the mRNA level of neighboring genes, using released data from GTEx. We found that rs6090311 G genotype was significantly associated with lower* BIRC7* (**Figure 1A**), *RP5-963E22.4* (**Figure 1B**), and* NKAIN4* (**Figure 1C**) mRNA levels when compared to rs6090311 A genotype.

## Discussion

The etiology of hepatoblastoma remains obscure. To identify the genetic profile and elucidate the pathogenesis of hepatoblastoma, we performed this case-control study including 313 cases and 1446 controls. Overall, we identified that the *YTHDF1* gene rs6090311 A>G polymorphism was associated with a decreased hepatoblastoma risk.

*YTHDF1* gene resides in chromosome 20q11. Several lines of evidence suggest the implication of YTHDF1 in tumorigenesis. Shi et al. 47 showed that YTHDF1 deficiency suppressed non-small cell lung cancer cell proliferation and xenograft tumor formation. However, they found that high expression of YTHDF1 was associated with better clinical outcomes. Zhao et al. 48 observed that upregulation of YTHDF1 was correlated with poor prognosis in patients with hepatocellular carcinoma. Han et al. 49 demonstrated that YTHDF1 regulated the anti-tumor immunity response via RNA m^6^A modification. Nishizawa et al. 50 reported that colorectal cancer patients with high YTHDF1 expression had significantly more reduced overall survival. Molecular mechanism experiments showed that c-Myc could drive YTHDF1 to promote cancer proliferation. The oncogenic role of YTHDF1 in colorectal cancer was also observed by Bai et al. 51.

Only one study has been conducted so far, regarding the epidemiology assessment of *YTHDF1* gene SNPs and cancer risk. Meng et al. 52 carried out a two-stage association study on colorectal cancer in China, consisting of the discovery stage with 1150 cases and 1342 controls, and the replication stage with 932 cases and 966 controls. They extensively genotyped 240 SNPs in 20 genes involved in m^6^A modification. Finally, the SNP rs118049207 located in the *SND1* gene was found to associate with colorectal cancer risk in the Chinese population. Further functional experiments suggested that rs118049207 could regulate mRNA expression of the *SND1* gene and then alter m^6^A level. Nevertheless, they failed to detect the association of SNPs rs2024768 and rs6090289 in the *YTHDF1* gene with colorectal cancer risk. In the current study, we assayed the genotype frequencies of two *YTHDF1* SNPs in a representative set of 313 hepatoblastoma cases and 1446 controls. No correlation between rs6011668 C>T and hepatoblastoma risk was observed, but hepatoblastoma risk was significantly decreased in participants with the rs6090311 G allele. Moreover, subjects carrying two protective genotypes showed significantly reduced hepatoblastoma risk when compared with those with 0-1 protective genotypes. FPRP analysis further validated the strength of the significant findings. These data indicate that *YTHDF1* gene SNPs are associated with hepatoblastoma susceptibility. We further attempted to interpret the possible mechanism of *YTHDF1* gene rs6090311 A>G-mediated hepatoblastoma risk. eQTL evidence suggested that the rs6090311 G allele is significantly associated with decreased *BIRC7*, *RP5-963E22.4*, and* NKAIN4* levels. Further functional experiments are needed to elucidate this mechanism.

Our study is strengthened by its size, multiple-center participants, and inclusion of strict genotyping quality control; however, some limitations should be addressed. Firstly, the sample size of our study was moderate. There is a possibility that some of the stratification analysis results might be merely fortuitous events. Secondly, all participants here are restricted to children of Chinese descent. Further studies are therefore warranted to delineate genetic contributions of* YTHDF1* to hepatoblastoma susceptibility in populations of non-Chinese ancestries. Thirdly, only two SNPs were analyzed in the current study, more SNPs are needed to be investigated in the near future. Finally, the current study only focused on genetic factors, and environmental factors were not considered in this study. The impact of *YTHDF1* gene SNPs is weak. This finding reinforces the notion that hepatoblastoma is a polygenic process and thus a combined analysis of multiple factors may have a greater ability to characterize this disease.

In all, we present the first epidemiology evidence supporting the involvement of *YTHDF1* gene polymorphisms in hepatoblastoma risk. Intensive future research will be needed to extend the discovery of hepatoblastoma susceptibility loci to individuals of non-Chinese ancestries.

## Supplementary Material

Supplementary figures and tables.

## Figures and Tables

**Table 1:** Association between *YTHDF1* polymorphisms and hepatoblastoma risk

Genotype	Cases (N=313)	Controls (N=1444)	*P*^ a^	Crude OR (95% CI)	*P*	Adjusted OR (95% CI) ^b^	*P*^ b^
**rs6011668 C>T (HWE=0.882)**							
CC	221 (70.61)	1047 (72.51)		1.00		1.00	
CT	83 (26.52)	366 (25.35)		1.07 (0.81-1.42)	0.614	1.08 (0.82-1.42)	0.603
TT	9 (2.88)	31 (2.15)		1.38 (0.65-2.93)	0.408	1.35 (0.63-2.87)	0.441
Additive			0.405	1.11 (0.87-1.40)	0.405	1.10 (0.87-1.40)	0.413
Dominant	92 (29.39)	397 (27.49)	0.497	1.10 (0.84-1.44)	0.497	1.10 (0.84-1.44)	0.495
Recessive	304 (97.12)	1413 (97.85)	0.433	1.35 (0.64-2.86)	0.435	1.32 (0.62-2.81)	0.469
**rs6090311 A>G (HWE=0.240)**							
AA	142 (45.37)	558 (38.64)		1.00		1.00	
AG	133 (42.49)	696 (48.20)		**0.75 (0.58-0.98)**	**0.032**	**0.75 (0.58-0.98)**	**0.033**
GG	38 (12.14)	190 (13.16)		0.79 (0.53-1.17)	0.231	0.79 (0.53-1.17)	0.240
Additive			0.066	0.84 (0.70-1.01)	0.066	0.84 (0.70-1.01)	0.070
Dominant	171 (54.63)	886 (61.36)	0.028	**0.76 (0.59-0.97)**	**0.028**	**0.76 (0.59-0.97)**	**0.029**
Recessive	275 (87.86)	1254 (86.84)	0.627	0.91 (0.63-1.32)	0.627	0.92 (0.63-1.33)	0.643
**Combined effect of protective genotypes ^c^**							
0	8 (2.56)	31 (2.15)		1.00		1.00	
1	135 (43.13)	527 (36.50)		0.99 (0.45-2.21)	0.986	1.01 (0.45-2.26)	0.976
2	170 (54.31)	886 (61.36)	0.026	0.74 (0.34-1.65)	0.465	0.76 (0.34-1.68)	0.497
0-1	143 (45.69)	558 (38.64)		1.00		1.00	
2	170 (54.31)	886 (61.36)	0.021	**0.75 (0.59-0.96)**	**0.021**	**0.75 (0.59-0.96)**	**0.022**

OR, odds ratio; CI, confidence interval; HWE, Hardy-Weinberg equilibrium. ^a^ χ^2^ test for genotype distributions between hepatoblastoma patients and cancer-free controls. ^b^ Adjusted for age and gender. ^c^ Protective genotypes were carriers with rs6011668 CC/CT, and rs6090311 AG/GG genotypes.

**Table 2:** Stratification analysis for association between *YTHDF1* genotypes and hepatoblastoma susceptibility

Variables	rs6011668 (case/control)		AOR (95% CI) ^a^	*P* ^a^	rs6090311 (case/control)		AOR (95% CI) ^a^	*P* ^a^	Combine genotypes (case/control)		AOR (95% CI) ^a^	*P* ^a^
	CC	CT/TT			AA	AG/GG			0-1	2		
**Age, month**												
<17	121/465	47/176	1.02 (0.70-1.49)	0.933	71/255	97/386	0.90 (0.64-1.28)	0.564	72/255	96/386	0.88 (0.63-1.25)	0.476
≥17	100/582	45/221	1.18 (0.80-1.74)	0.395	71/303	74/500	**0.63 (0.44-0.91)**	**0.012**	71/303	74/500	**0.63 (0.44-0.91)**	**0.012**
**Gender**												
Female	99/427	30/168	0.77 (0.49-1.21)	0.255	60/236	69/359	0.76 (0.52-1.11)	0.151	60/236	69/359	0.76 (0.52-1.11)	0.151
Male	122/620	62/229	1.37 (0.97-1.93)	0.072	82/322	102/527	0.77 (0.56-1.06)	0.105	83/322	101/527	0.75 (0.54-1.03)	0.080
**Clinical stages**												
I+II	116/1047	44/397	1.00 (0.69-1.44)	0.997	73/558	87/886	0.75 (0.54-1.05)	0.092	73/558	87/886	0.75 (0.54-1.05)	0.092
III+IV	63/1047	28/397	1.17 (0.74-1.86)	0.496	41/558	50/886	0.77 (0.50-1.17)	0.218	42/558	49/886	0.73 (0.48-1.12)	0.150

AOR, adjusted odds ratio; CI, confidence interval; ^a^ Adjusted for age and gender, omitting the corresponding stratify factor.

**Table 3:** False-positive report probability values for the associations between *YTHDF1* gene polymorphisms and hepatoblastoma susceptibility

Genotype	Crude OR (95% CI)	*P* ^a^	Statistical Power ^b^	Prior probability				
				0.25	0.1	0.01	0.001	0.0001
**rs6090311 A>G**								
AG vs. AA	0.75 (0.58-0.98)	0.032	0.847	**0.101**	0.252	0.787	0.974	0.997
AG/GG vs. AA	0.76 (0.59-0.97)	0.028	0.840	**0.091**	0.230	0.767	0.971	0.997
≥17	0.63 (0.44-0.90)	0.011	0.377	**0.082**	0.212	0.748	0.968	0.997
**Protective genotypes**								
2 vs. 0-1	0.75 (0.59-0.96)	0.021	0.814	**0.073**	**0.191**	0.722	0.963	0.996
≥17	0.63 (0.44-0.90)	0.011	0.377	**0.082**	0.212	0.748	0.968	0.997

OR, odds ratio; CI, confidence interval. ^a^ Chi-square test was used to calculate the genotype frequency distributions. ^b^ Statistical power was calculated using the number of observations in the subgroup and the OR and *P* values in this table.
