# Supplementary material for: YTHDF1 rs6090311 A>G polymorphism reduces Hepatoblastoma risk: Evidence from a seven-center case-control study
Source: J Cancer. 2020 Jun 28;11(17):5129–34. doi: 10.7150/jca.46120 (PMC7378914; doi:10.7150/jca.46120)
Supplement: Supplementary file 1 — Supplementary figures and tables. [file jcav11p5129s1.pdf]

Table S1. Frequency distribution of selected variables in hepatoblastoma patients and cancer-free controls

Variables	Cases (N=313)		Controls (N=1446)		P^a
	No.	%	No.	%	
Age range, month	0.03-149.97		0.004-156.00		
Mean ± SD	23.75 ± 25.93		25.23 ± 19.38		0.251[b]
<17	168	53.67	642	44.40	
≥17	145	46.33	804	55.60	
Gender					0.983
Female	129	41.21	595	41.15	
Male	184	58.79	851	58.85	
Clinical stages					
I	97	30.99	/	/	
II	63	20.13	/	/	
III	64	20.45	/	/	
IV	27	8.63	/	/	
NA	62	19.81	/	/	

SD, standard deviation, NA, not available.

[a] Two-sided χ^2 test for distributions between hepatoblastoma cases and cancer-free controls.

[b] T-test for age distribution between hepatoblastoma patients and cancer-free controls.

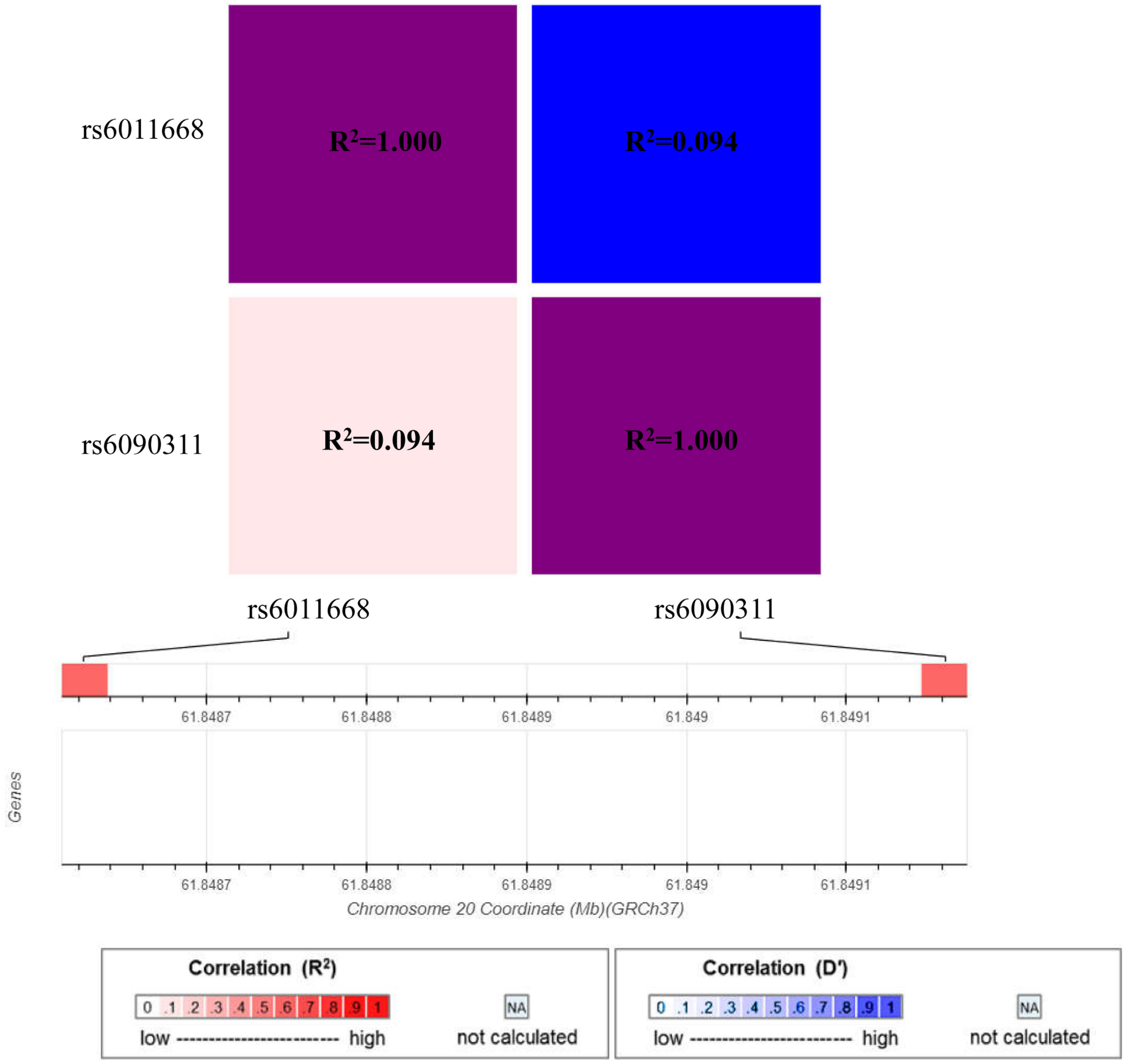

Figure S1. Linkage disequilibrium (LD) analysis for the two selected SNPs in Chinese Han population consisting of CHB (Han Chinese in Beijing, China) and CHS (Southern Han Chinese) subjects. LD as R^2 for SNP pairs is shown inside the squares.
